# Measuring Noseband Tightness on the Lateral Aspect of the Horse’s Face

**DOI:** 10.3390/ani15040537

**Published:** 2025-02-13

**Authors:** Russell MacKechnie-Guire, Hilary Clayton, Jane Williams, David Marlin, Mark Fisher, Diana Fisher, Victoria Walker, Rachel Murray

**Affiliations:** 1 Equine Department, Hartpury University, Hartpury, Gloucester GL19 3BE, UK; jane.williams@hartpury.ac.uk (J.W.); victoria.walker@hartpury.ac.uk (V.W.); 2 Department of Large Animal Clinical Sciences, College of Veterinary Medicine, Michigan State University, 736 Wilson Road, East Lansing, MI 48824, USA; claytonh@msu.edu; 3 Animalweb Ltd., The Granary, Hermitage Court, Hermitage Lane, Maidstone, Kent ME16 9NT, UK; dm@davidmarlin.co.uk; 4 Woolcroft Equine Services, May Lane, Wisbech PE13 5BU, UK; woolcroft2002@yahoo.co.uk (M.F.); dianafisher007@yahoo.co.uk (D.F.); 5 Ibikus Ltd., Bury St Edmunds IP32 7AR, UK; rmurray@ibikus.co.uk

**Keywords:** lateral, bridle fit, nasal, cavesson, Swedish (crank), surface, anatomical, welfare

## Abstract

Measuring tools designed to objectively determine equine noseband tightness are inserted on the dorsal nasal planum in a rostro-caudal direction. The lateral aspect of the horse’s head has several areas where minimal soft tissue intervenes between the skin and underlying bone, which makes them potentially useful sites for measuring noseband tightness. One hundred horses were fitted with a snaffle bridle with a cavesson, Swedish or dropped noseband in random order. The tightness of each noseband type was adjusted sequentially to 2.0, 1.5, 1.0, 0.5 and 0.0 finger-equivalents using an ISES Taper Gauge. For each adjustment, a digital calliper determined the distance (mm) between the inner surface of the noseband at three lateral locations: (1) lateral nasal bone, (2) lateral maxilla rostral to the facial crest, and (3) lateral mandible. Friedman’s analysis was used to test the differences between locations (*p* < 0.02). No differences were found between 2.0 and 1.5 finger-equivalent tightness at the nasal and maxillary sites for the cavesson (*p* = 0.89, *p* = 0.03, respectively) and Swedish (*p* = 0.06, *p* = 0.40, respectively) noseband. When adjusted between 2.0 to 0.5 finger-equivalent tightness, the coefficient of variation was “good” for the nasal (16%) and maxilla (19%) sites. These results indicate that a lateral measuring site may provide a suitable addition to dorsal midline measurements.

## Simple Summary

Measuring tools to determine noseband tightness are available but their use requires insertion beneath the noseband on the dorsal nasal bone where the noseband is fitted. There are several locations on the lateral aspect of the head that may be practical and repeatable, offering additional sites. This study investigated the repeatability of using three anatomical locations on the lateral aspect of the horse’s head to determine noseband tightness. One hundred horses were recruited and fitted with a snaffle bridle with a cavesson, Swedish (crank) or dropped noseband. Using an ISES Taper Gauge, noseband tightness was adjusted for each noseband (2.0 to 0.0 finger-equivalents). For each adjustment, a digital calliper determined the distance (mm) between the inner surface of the noseband and three lateral locations on the nasal bone, the maxilla and the mandible. Friedman’s analysis was used to test differences between locations (adjusted significance *p* < 0.02). At 2.0 and 1.5 finger-equivalent tightness, the coefficient of variation was “good” for the lateral nasal and maxillary sites. These sites could potentially be used as locations in addition to the dorsal nasal site for determining noseband tightness. These data can be used to inform the dimensions of a laterally admitted measuring tool.

## 1. Introduction

The *Federation Equestre Internationale* (FEI) and national equestrian federations have rules to protect the health and welfare of horses during competitions. These include regulations that identify suitable tack and equipment that can be worn and how these should be fitted. Traditionally, it is recommended that a horse’s noseband should be adjusted to allow two human fingers to be admitted beneath the noseband (British Horse Society), a method commonly adopted/followed by riders in the UK and Northern and Central America [1,2] and Europe [3]. Where this guidance originates from is unknown and it does not appear to be based on empirical data. The two-finger method has challenges because finger dimensions vary [4]. Furthermore, it is unclear if this method is based on two fingers positioned side by side [4] or in a stacked formation [5]. The exact location where the measurement should be taken also varies among users and countries, with two fingers being inserted beneath the noseband on the dorsal nasal bone or the lateral aspect of the horse’s head. FEI guidelines relating to the measurement location state: *“It must be possible to place at least two fingers between the horse’s cheek and the noseband. Both fingers are to be placed side by side, flat against the horse’s cheek” (FEI rules and regulations, accessed 2024).*

In national/international dressage and eventing competitions (2017), measurements of 750 horses found that 44% of the nosebands had been tightened to zero fingers, and only 7% of nosebands complied with the two-finger recommendation [6]. More recently (2019), 59% of Dutch riders had their noseband adjusted according to the two-finger regulation [7]. This higher percentage of nosebands adjusted to two finger-equivalents may reflect a greater awareness amongst stakeholders of the importance of correct noseband fit. Competitors were in support of rules that standardise noseband tightness (74%) and agreed that using a measuring tool is a fair method to determine tightness (68%), although slightly fewer respondents agreed that the noseband fit should be measured on the dorsal aspect of the nasal bone (65%) [2].

The adoption of an objective measuring tool across equestrian competition would help safeguard the horses’ welfare by preventing excessively tight nosebands, as well as providing a mechanism for stewards to check compliance with noseband tightness rules. Measuring tools that provide a standardised method of determining the tightness of the noseband are available [4,5]. Based on the finger dimensions of 10 female and male subjects, The International Society of Equitation Science (ISES) designed a non-deformable measuring tool that equates to the dimensions of two fingers (6.08 cm^2^) positioned in a side-by-side orientation and measured at the midpoint of the middle phalanges of the second and third digits: width: 3.87 ± 0.09 cm, circumference: 9.89 ± 0.21 cm [4]. The tool has visual indicators representing 2.0 and 1.0 finger-equivalent tightness. The inability to insert the measuring tool to the relevant indicator confirms that the noseband tension exceeds the intended tightness.

Based on the dimensions of female fingers, arranged in a stacked formation of less than two, two, or more than two fingers, a multi-tool has been designed and used to measure noseband tightness in a large number of Danish competition horses [5]. Like the ISES tool, the multi-tool is inserted between the nasal bone and the noseband in a rostro-caudal direction, with the tightness being converted to a linear measurement (<2, 2 and >2 cm) [5]. Recently (October 2024), the FEI has designed a measuring tool similar to existing tools that is inserted mid-dorsally but in a caudal-rostral direction. The dimensions of the tool (4.67 cm^2^) have been informed by data [8,9] and have been tested on over six hundred horses in Europe and America (https://inside.fei.org, Veterinary Rules and Regulations, accessed 1 November 2024). If the noseband is adjusted to the correct level of laxity, the tool should pass beneath it; however, if the noseband is too tight (<1.5 finger-equivalent tightness), the tool will not pass through, resulting in a failed test. With effect from May 2025, the FEI will implement the new measuring tool for tack and equipment checks, with the overarching objective of safeguarding horses from tight nosebands (https://inside.fei.org, Veterinary Rules and Regulations, accessed 1 November 2024).

Despite tools being available for over ten years, their use has not been widely implemented. One of the limitations voiced by industry stakeholders of the existing tools is that they are designed to be used beneath the noseband on the dorsal nasal planum, inserted in a rostro-caudal direction. This location is used due to the lack of underlying compressible soft tissues, but it is an area subject to considerable anatomical variation [10,11]. In a recent study, 84% of horses scored 7 or above on a comfort scale, where 10 was defined as extremely comfortable and cooperative when noseband tightness was being assessed [4]. In a recent FEI field study in competition, 600 horses were tested with only a few horses objecting to the tool being admitted beneath the noseband (https://inside.fei.org, Veterinary Rules and Regulations, accessed 1 November 2024).

However, although measuring tools have been successfully used [2,5,6,7,9], additional locations for noseband measurement have not been investigated. The first criterion for a suitable anatomical location is the presence of minimal soft tissues between the skin and the underlying bone. Individual variations in equine head anatomy influence the forces beneath the noseband [10,11]. A noseband comprises a length of material (generally leather or leather-like material) that encircles the horse’s face rostral to the facial crest [6] and is fastened by a buckle or other attachment ventral to the horizontal rami of the mandible. The cavesson and flash nosebands are most widely used in competition and training [1,2,5,6]. The Swedish noseband is most often used in dressage [7]. The drop noseband is also used but, due to its rostral position, might not be amenable to the use of all measuring tools.

Ideally, a measuring tool for measuring noseband tightness should be able to be used with many different nosebands; be non-deformable; be horse/handler friendly in terms of shape, design and colour; and be inserted at a convenient location that provides repeatable measurements [12]. We identified three easily identifiable locations beneath the noseband on the lateral side of the skull that fulfil these requirements. The first location is over the lateral aspect of the nasal bone close to or over the nasomaxillary suture, the second is over the maxilla rostral to the upper edge of the facial crest, and the third location is on the lateral aspect of the mandible.

The objectives of this study were to determine the separation of the noseband from the horse’s head at three anatomical locations (lateral nasal bone, lateral maxilla rostral to the facial crest and lateral mandible) beneath a noseband adjusted to 2.0, 1.5, 1.0, 0.5 or 0.0 finger-equivalent laxity measured mid-dorsally using an ISES taper gauge, and to compare the repeatability of measurements at all lateral locations. It was hypothesised that 1) noseband separation from the head would decrease with each tightness adjustment at all lateral locations and 2) the three measuring locations would have excellent (<10%) to good (10–20%) coefficients of variation (CoV).

## 2. Materials and Methods

### 2.1. Ethics

Ethics were approved by Hartpury University’s Ethics Committee: URN 2023-125. As the noseband was fitted to 0.0 finger-equivalent tightness (defined as the ISES tool not being able to be admitted beneath the noseband, and no compression of the soft tissues), written confirmation that the study did not require regulation under the Animals Scientific Procedures Act 1986 was provided by the Home Office, United Kingdom (April 2024). Informed, written consent was obtained from riders and owners prior to participation, and they were advised that they could withdraw from the study at any point. The trial was terminated if the horse became unsettled or demonstrated undesirable behaviour.

### 2.2. Intra and Inter-Assessor Repeatability

**Figure 1:**
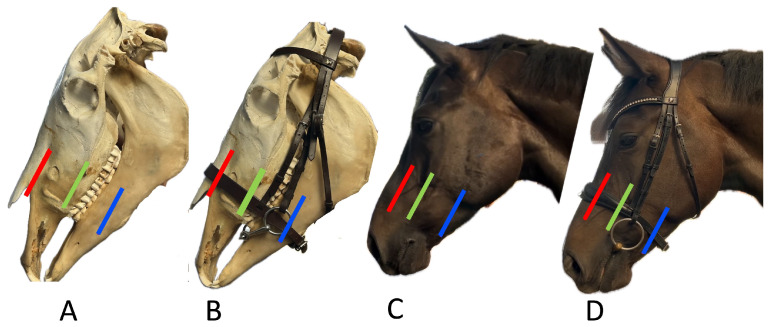
Illustration of the three lateral locations: lateral aspect of the nasal bone close to or over the nasomaxillary suture (red line), lateral maxilla rostral to the upper edge of the facial crest (green line) and lateral aspect of the mandible (blue line) on a skull (**A**), skull fitted with a cavesson noseband (**B**), a horse with no bridle (**C**) and a horse fitted with a cavesson noseband (**D**).

To safeguard the horse, a model horse fitted with a snaffle bridle and cavesson noseband was used to train seven equine professionals (3 coaches, 2 researchers, 2 professional riders) in the theory and practice of measuring the distance between the inner surface of a cavesson noseband and the model horse’s head for all noseband tightness adjustments (2.0 to 0.0 finger-equivalents tightness) at the three anatomical locations (Figure 1). Three repeated measurements were taken at each location for each noseband, and the CoV was calculated for each noseband tightness. Using thresholds described by Aronhime et al. [12], the CoV (%) was defined as excellent (<10%), good (10–20%), acceptable (20–30%) or poor (>30%).

Using three horses (mean ± S.D: 13 ± 1 years, 162 ± 4.0 cm), three assessors (1 qualified saddle fitter (25 years’ experience) and 2 equine researchers (20 years’ experience) were provided with theoretical and practical training in advance and on the day of data collection. Training included how to identify the three measurement locations on the live horse and how to use the digital gauge. To determine variations between measurements and the three assessors, a repeatability study was performed whereby the three assessors measured the distance between the inner surface of a cavesson and a Swedish noseband at all noseband tightness adjustments (2.0 to 0.0 finger-equivalents tightness) at the three anatomical locations evaluated. Three repeated measurements were taken at each location for each noseband, and the CoV was calculated for each noseband and tightness.

### 2.3. Horses

After the repeatability study was completed, one hundred horses were recruited: Warmbloods (n = 24), Thoroughbreds (n = 6), Irish Sport Horses (n = 65) and Spanish (n = 5) horses. Horses were aged (mean ± S.D) 10 ± 3.5 years and height 164 ± 4.3 cm. All horses were actively participating in dressage, show jumping, eventing or military service (The Kings Troop, Royal Horse Artillery). As part of the inclusion criteria, horses were required not to display negative behaviour related to the bit or bridle (e.g., biting, head shaking, head shy) when being handled or when the bridle was being fitted or removed. At some stage in the horse’s training career, all horses had worn each noseband type. The horses were usually trained in a Swedish noseband (n = 40), a cavesson (n = 40), or a dropped noseband (n = 20) adjusted to a two finger-equivalent tightness.

### 2.4. Bridles

Five identical experimental bridles (Passier™: 2 cob, 2 full and 1 extra full size), all featuring a shaped headpiece, two cheek pieces and a throat lash were prepared. A bridle was fitted to each horse with the horse’s own bit (76 loose ring and 24 eggbutt snaffles, 12.0–15.2 cm wide) by two experienced (four years) qualified bridle fitters (Society of Master Saddlers).

Each horse was fitted with:

- Cavesson noseband: a single band encircling the nose 2–4 cm rostral to the facial crest, fastened with a buckle beneath the mandible, with the front part of the band lined by simple padding 4 mm thick.
- Swedish (crank) noseband: dorsally similar to a cavesson with 4 mm thick padding over the nasal bones. The ventral strap passed through two D rings and was adjusted to position its pad (thickness: 4 mm) over the mandibular rami.
- Dropped noseband: positioned further rostrally with the front of the noseband positioned dorsal to the nasal bones and its ventral strap passing below the bit and sitting within the chin groove.

### 2.5. Noseband Tightness

The ISES taper gauge [4] was inserted mid-dorsally beneath the noseband strap in a rostro-caudal direction until noseband tension resisted further movement. The marks indicating two finger-equivalents (40 mm wide × 16 mm depth) and one finger-equivalent (18 mm wide × 11 mm depth) were used. Additional horizontal lines were added between these to represent 0.5 (16 mm wide × 10 mm depth) and 1.5 (30 mm wide × 15 mm depth) finger-equivalent tightness. Zero finger-equivalent tightness was determined by the inability to admit the taper gauge beneath the noseband without compression of the skin and soft tissues beneath the noseband. Using the ISES taper gauge, the same researcher adjusted each noseband type to the required tightness levels; the corresponding setting (hole) on the noseband was then recorded by a research assistant.

### 2.6. Lateral Head Measurements

**Figure 2:**
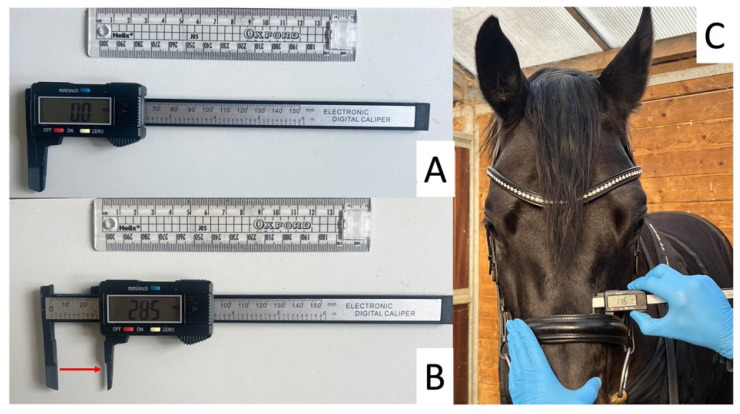
Illustration of (**A**) the digital gauge in a closed position (10 mm width); (**B**) the digital gauge in a position where the mobile arm has been retracted, as indicated by the red arrow; and (**C**) the digital gauge being used to determine the distance between the noseband and the lateral nasal location. The digital display displays the distance between the two arms in mm.

For each horse, at each noseband tightness (2.0, 1.5, 1.0, 0.5 and 0.0 finger-equivalents tightness), a modified calibrated digital gauge (Kynup, measuring range 0–150 mm, resolution 0.01 mm and accuracy ±0.01 mm) was used to measure the distance between the inner surface of the noseband at the lateral nasal, maxillary and mandibular locations (Figure 2). To obtain the measurements, the digital gauge was carefully inserted in a caudal-rostral direction beneath the lateral aspect of the noseband at each of the three locations. With the researcher standing on the left side of the horse, the digital gauge was inserted so that the outer aspect of the stationary post of the calliper lay against the lateral aspect of the horse’s head. The researcher applied pressure to retract the mobile branch until the noseband resisted any further retraction. The gauge was positioned to ensure that the extending arm was perpendicular to the measuring location (Figure 2). With the horse standing still and its neck in a horizontal position at the height of the withers and their nasal bone close to the vertical, three measurements were taken at each location, with the digital output being recorded by a research assistant. The researcher was blinded to the measurements and only looked at the digital output when the measurement was completed. The trial was terminated if the horse initiated a chewing cycle or became unsettled or displayed behaviour indicative of discomfort: for example, persistent head raising/lowering, stepping forwards/backwards/sideways, left/right front limb pawing, biting and teeth grinding. If a horse’s behaviour was felt to potentially compromise the safety or welfare of the horse or the measuring team, the trial was terminated.

### 2.7. Study Design

Horses were randomly assigned to one of the three assessors. Assessors were asked to rank the three sites according to the ease of measurement and the horse’s compliance. Bridle fit, noseband adjustments and measurements were performed with the horse standing in its own stable or stall. A handler was positioned on the right side of the horse, and the assessor performed all noseband measurements when standing on the left side of the horse. Noseband type was assessed in a random order and tightness was assessed from loosest to tightest. Three measurements were taken with the digital gauge at each location.

### 2.8. Data Analysis

The Kolmogorov–Smirnov test indicated that the data distribution was non-parametric. Therefore, a Friedman’s 2-way ANOVA was used to detect differences between noseband tightness levels at each anatomical location. Where significant differences were found, subsequent post hoc Wilcoxon signed-rank tests identified where differences occurred between noseband types and tightness. As multiple comparisons were performed, a Bonferroni adjustment was applied, resulting in an adjusted alpha of *p* ≤ 0.02.

### 2.9. Receiver-Operator Characteristic (ROC)

ROC analyses are often used in clinical studies to determine and test the accuracy or predict diagnostic thresholds that determine whether patients have a disease or to test the precision of medical tests [13].

Previously, in a group of high-level dressage horses trotting with small pressure mats beneath the noseband, we found no significant differences in noseband pressures when the noseband was adjusted at 2.0 and 1.5 finger-equivalent tightness, compared to the tighter adjustments (1.0, 0.5 and 0.0 finger-equivalent tightness), where noseband pressures were increased [9]. Therefore, these noseband tightening settings (2.0 and 1.5 finger-equivalents) were selected to evaluate if a predictive threshold measure could be determined for noseband tightness from the data collected. Median values for noseband tightness across all horses and noseband types for the 2.0 and 1.5 finger-equivalent tightness were calculated at the maxillary site (30.2 mm and 27.2 mm, respectively) and lateral nasal site (29.3 mm and 26.2 mm, respectively) as the data were non-parametrically distributed to determine a central measure to be used as a threshold to assign an initial pass/fail value for noseband tightness to 1.5 and 2.0 finger-equivalent tightness measurements for the cohort to support ROC analysis. Receiver-operator characteristic (ROC) curves were plotted to determine the ability of the assigned threshold measures to predict a ‘pass’ outcome to assess noseband tightness suitability when measured at lateral nasal and maxillary locations [14,15]. Measurements taken were then assigned as a pass if they exceeded these thresholds or a failure result if below them.

For the ROC curves, the area under the curve (AUC) values were interpreted to assess the predictability of the assigned threshold values to determine if noseband tightness was above or below the assigned threshold. This was determined by the assessment of the AUC value; median values of AUC < 0.5 suggest no discrimination, i.e., the threshold is not accurate; AUC 0.7 to 0.8 suggest the threshold used has acceptable discrimination, i.e., the threshold has acceptable reliability; AUC 0.8 to 0.9 is excellent, i.e., the threshold has excellent reliability, and AUC > 0.9 has outstanding reliability [16,17]. Sensitivity and specificity coordinates were also plotted and examined to identify a more accurate threshold level for future measurements, using 90% (>0.90) as a minimum value for both sensitivity and specific coordinates. These values were used to calculate positive and negative predictive values for measurements [18] using the following formulae:

$$\begin{array}{l} Positive\;Predictive\;Value\left(PPV\right)\\ =\frac{\left(sensitivity\times prevalence\right)}{\left(sensitivity\times prevalence)+((1-specificity)\times (1-prevalence)\right)} \end{array}$$

$$\begin{array}{l} \text{Negative}\;\text{Predictive}\;\text{Value}\;\left(NPV\right)\\ =\frac{\left(specificity)\times (1-p\right)}{\left(specificity)\times (1-p)+((1-sensitivity)\times (p)\right)} \end{array}$$

The PPV determines the percentage of subjects who record a true positive value in a test, i.e., how many record a positive/pass or how accurate a specific test is to determine the likelihood that a person has a specific disease or as here, how accurate a threshold measure is for a test to be assigned accurately as a pass for noseband tightness. The NPV identifies the percentage of subjects who record a true negative value in a test, i.e., how many record a negative/fail or how accurate a specific test is to determine the likelihood that a person does not have a specific disease, or as here, how accurate a threshold measure is for a test to not be assigned accurately as a pass for noseband tightness.

## 3. Results

### 3.1. Inter-Assessor Repeatability Results

For the model horse fitted with a cavesson noseband, the inter-assessor CoV for the lateral nasal and maxillary measurements at all noseband adjustment levels was excellent (≤9%). For the lateral mandible, when the noseband was adjusted to 2.0 finger-equivalent tightness, the inter-assessor CoV was good (10%) and for the remaining adjustments was excellent (≤5%). When horses were fitted with a cavesson or Swedish noseband, the intra-assessor CoV was excellent (≤8%) for all tightness adjustments (2.0 to 0.0 finger-equivalents) at all three locations [12] (Table 1).

### 3.2. Horses

Three horses were withdrawn from the study due to persistent head raising/lowering when the ISES taper gauge was inserted beneath the noseband at 2.0 finger-equivalent tightness. One horse was withdrawn due to persistent head raising/lowering/turning when the lateral measurements were taken at the lateral nasal and maxillary sites. Measurements of noseband separation from the face at the lateral nasal, maxillary and mandibular sites were obtained from 96 horses with the noseband adjusted to 2.0 and 1.5 finger-equivalent tightness.

Six horses were removed from the tighter noseband adjustments (1.0, 0.5 and 0.0 finger-equivalents) due to persistent head raising and lowering when the ISES taper gauge was admitted, and four horses were withdrawn from the tighter adjustments at the request of the owner due to sensitivity relating to noseband tightness, resulting in 86 horses being included in the 1.0, 0.5 and 0.0 finger-equivalents analysis. When the noseband was adjusted to 0.0 finger-equivalents, the digital gauge (in the closed position: 10 mm width) could not be inserted beneath the noseband at any lateral location in 51% of 86 horses.

The dropped noseband was included in 25 horses, but the lateral locations could not be evaluated because the anatomical landmarks were not present in the more rostral position of this noseband.

### 3.3. Differences in the Distance Between the Anatomical Locations and the Inner Part of the Noseband

No significant differences were found in the distance (mm) that the noseband was separated from the lateral nasal and maxillary sites for the Swedish noseband at any adjustment from 2.0 to 0.0 finger-equivalent tightness (all *p* ≥ 0.03). For the cavesson noseband, there were no significant differences in noseband separation between the lateral nasal and maxillary sites when adjusted to 2.0 (*p* = 0.89), 1.5 (*p* = 0.03) and 0.0 (*p* = 0.12) finger-equivalent tightness. When adjusted to 1.0 and 0.5 fingers equivalent tightness, noseband separation was greater for the maxillary vs. nasal site (*p* = 0.008, *p* = 0.01, respectively).

**Figure 3:**
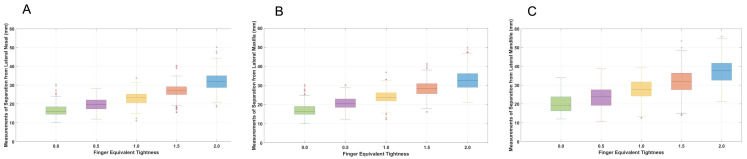
Boxplots illustrating data for cavesson noseband adjustments from 2.0 to 0.0 finger-equivalent tightness at each anatomical location on the lateral aspect of the horse’s head: lateral nasal (**A**), maxillary (**B**) and mandibular (**C**). The central line represents the median; the box represents the 25th and 75th percentile; and the whiskers represent the maxima and minima not considered outliers. + represents outliers.

**Figure 4:**
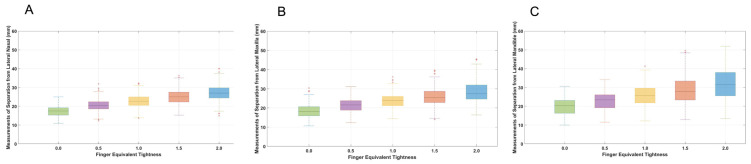
Boxplots illustrating data for Swedish noseband adjustments from 2.0 to 0.0 finger-equivalent tightness for each anatomical location on the lateral aspect of the horse’s head: lateral nasal bone (**A**), maxilla (**B**) and mandible (**C**). The central line represents the median; the box represents the 25th and 75th percentile; and the whiskers represent the maxima and minima, not considered outliers. + represents outliers.

**Figure 5:**
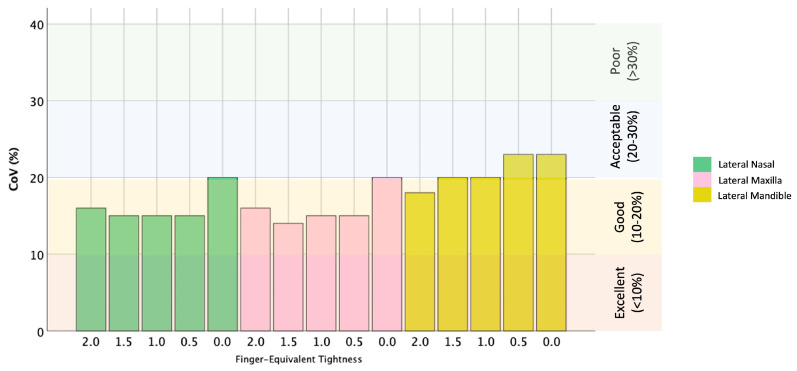
Coefficient of variation (CoV) (%) for the cavesson noseband across all tightness adjustments at the lateral nasal (green bars), maxillary (pink bars) and mandibular (yellow bars) sites. Previously published levels of agreement [12] are shown on the right of the graph.

**Figure 6:**
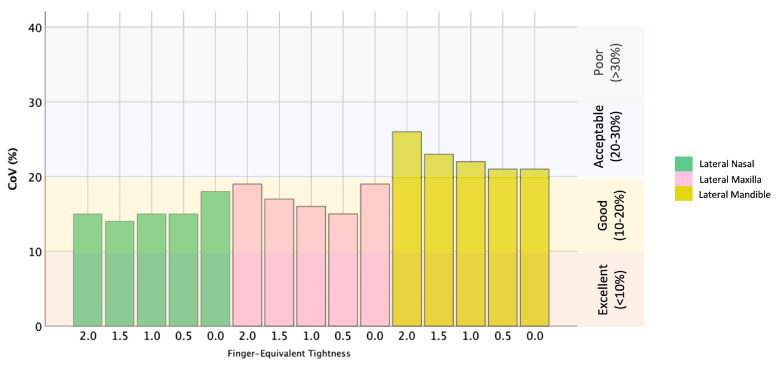
Coefficient of variation (CoV) (%) for the Swedish noseband across all tightness adjustments at the lateral nasal (green bars), maxillary (pink bars) and mandibular (yellow bars) sites. Previously published levels of agreement [12] are shown on the right of the graph.

For all cavesson noseband adjustments, there was greater separation at the mandibular site compared with the nasal and maxillary sites (both *p* < 0.001). For all Swedish noseband adjustments, noseband separation was greater for the mandibular site compared with the nasal site (*p* ≤ 0.003), but there was no difference between the maxillary and mandibular sites (*p* ≥ 0.06) (Figure 3 and Figure 4). The CoV for the cavesson (Figure 5) and Swedish (Figure 6) nosebands was predominantly “good” for the lateral nasal and maxillary sites in the range of 2.0 to 0.5 finger-equivalent tightness.

### 3.4. Noseband Separation for Each Tightness Adjustment

There was significantly less noseband separation at the lateral nasal site (*p* ≤ 0.001) when the cavesson noseband was adjusted from 2.0 to 1.5 (*p* = 0.008), 1.5 to 1.0 (*p* = 0.001), 1.0 to 0.5 (*p* = 0.01) and 0.5 to 0.0 (*p* = 0.01) finger-equivalent tightness. For the maxillary and mandibular sites, the noseband separation distance decreased for all noseband adjustments (*p* ≤ 0.001) except at 0.5 to 0.0 finger-equivalent tightness, where there was no difference *p* ≥ 0.02 (Figure 3).

When the Swedish noseband was adjusted from 2.0 to 1.5 finger-equivalent tightness, there was no difference in noseband separation for the lateral nasal (*p* = 0.09), maxillary (*p* = 0.11) and mandibular (*p* = 0.44) sites. For all remaining noseband adjustments (1.0 to 0.0 finger-equivalent tightness), the distance between the noseband and each anatomical site decreased as noseband tightness increased (*p* ≤ 0.01) (Figure 4).

### 3.5. Differences Between Noseband Types at Each Anatomical Location

**Figure 7:**
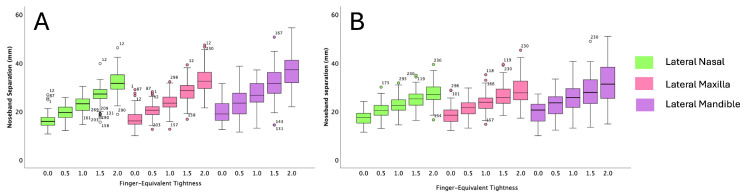
Boxplots illustrating data for cavesson (**A**) and Swedish (**B**) noseband adjustments from 0.0 to 2.0 finger-equivalent tightness for each anatomical location on the lateral aspect of the horse’s head: nasal bone (green boxes), maxilla (pink boxes) and mandible (purple boxes). The central line represents the median; the box represents the 25th and 75th percentile; and the whiskers represent the maxima and minima, not considered outliers. + represents outliers.

At 2.0 and 1.5 finger-equivalent tightness, the Swedish noseband had significantly less separation from the face than the cavesson noseband at the lateral nasal bone (both *p* < 0.001), maxilla (both *p* < 0.001), and mandible (both *p* < 0.001) (Figure 7). At 1.0 finger-equivalent tightness, the noseband separation from the face at the lateral mandible was less for the Swedish than the cavesson noseband (*p* = 0.005). At 0.5 and 0.0 finger-equivalent tightness, the Swedish noseband had significantly greater separation than the cavesson at the lateral nasal bone (*p* = ≤0.02, *p* = 0.01, respectively) and maxilla (both *p* = 0.01) (Figure 7).

### 3.6. Horse Comfort with Measurement Location

When the ISES taper gauge was used to measure the tightness of the cavesson and Swedish noseband, 46% of horses (n = 40) displayed behaviours that included raising/lowering the head (n = 33), stepping forwards/backwards/sideways (n = 20), left/right front limb pawing (n = 10) and teeth grinding (n = 5). For the dropped noseband, 60% of horses (n = 15) displayed aversive behaviours when using the ISES taper gauge to determine noseband tightness. For the calliper measurements, 10% of horses (n = 10) displayed aversive behaviour at the lateral nasal site when the noseband was tighter than 1.0 finger-equivalents.

### 3.7. Assessor Measurement Location Preferences

When the three assessors ranked the ease of obtaining data from the three lateral measurements, the maxilla was the preferred location in 80% of the studied horses, followed by the lateral nasal bone. Unanimously, all assessors reported that the mandibular site was the least preferred location.

### 3.8. Receiver-Operator Characteristic ROC

#### 3.8.1. Lateral Nasal

Both the median threshold for 1.5 and 2.0 finger-equivalent tightness demonstrated acceptable ROC AUC values (0.7–0.8) for discrimination across the cohort for lateral nasal measurements of noseband tightness (Table 2, Figure 7) again suggesting the values are suitable for determining a pass test for noseband tightness. Values of 17.8 mm (100% sensitivity, 90.6% specificity) and 20.7 mm (2.0 finger-equivalent tightness; 100% sensitivity, 90.9% specificity) were estimated to be the minimum threshold for predictability of suitable noseband tightness from 1.5 finger-equivalent tightness pass/fail results. Values of 18.9 mm (96.1% sensitivity, 90.9% specificity) and 20.7 mm (2.0 finger-equivalent tightness; 100% sensitivity, 90.9% specificity) were estimated to be the minimum threshold for predictability of suitable noseband tightness from the 2.0 finger-equivalent tightness pass/fail results.

#### 3.8.2. Lateral Maxilla

Both the median threshold for 1.5 and 2.0 finger-equivalent tightness demonstrated acceptable ROC AUC values (0.7–0.8) for discrimination across the cohort for maxillary measurements of noseband tightness across all noseband types (Table 2, Figure 6) suggesting the values are suitable for determining a pass test for noseband tightness. Predictive threshold values of 20.4 mm (1.5 finger-equivalent tightness; 100% sensitivity, 90.3% specificity) and 21.2 mm (2.0 finger-equivalent tightness; 100% sensitivity, 90.3% specificity) were estimated to be the minimum threshold for predictability of suitable noseband tightness from 1.5 finger-equivalent tightness pass/fail results. Predictive threshold values of 20.4 mm (1.5 finger-equivalent tightness; 100% sensitivity, 90.2% specificity) and 21.2 mm (2.0 finger-equivalent tightness; 100% sensitivity, 90.2% specificity) were estimated to be the minimum threshold for the predictability of suitable noseband tightness from 2.0 finger-equivalent tightness pass/fail results.

**Figure 8:**
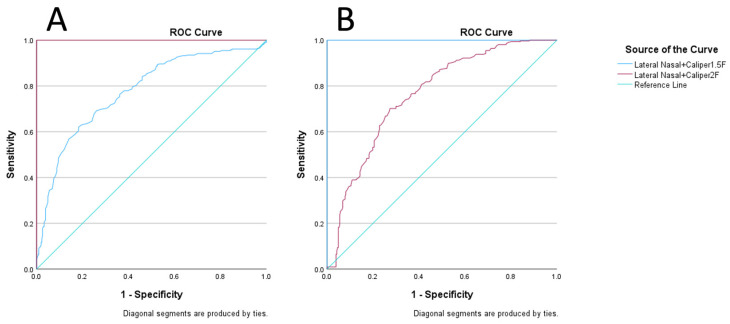
Receiver operator characteristic (ROC) curves: Predictability of 2.0 finger-equivalent tightness (**A**) 1.5 finger-equivalent tightness median threshold lateral nasal 1.5 and (**B**) 2.0 finger-equivalent tightness measurements across all noseband types.

The positive predictive values for all measures are high, suggesting the proposed thresholds discriminate well for true positive cases; however, the low negative predictive values also indicate the thresholds cannot differentiate a false negative case well (Figure 8).

## 4. Discussion

This study investigated the feasibility of assessing noseband tightness by measuring noseband separation from the face at three sites on the lateral aspect of the head. The nasal bone, maxilla and mandible were chosen because they were easy to identify and there was minimal soft tissue intervening between the skin and underlying bone, thus providing a rigid underlying surface for the measurements. The mandibular site was rated as the most difficult to access and the values recorded at that site were influenced by the chin pad on the Swedish noseband. The other two sites were easy to access, but about 10% of horses showed resistant behaviour when tested at the nasal site with ≤1.0 finger-equivalents tightness, whereas none were resistant to testing over the maxilla, which was also the preferred site for the assessors for a majority of horses.

These results have provided quantitative data about the repeatability and variation of noseband separation measurements at different adjustments from 2.0 to 0.0 finger-equivalents. The first hypothesis that noseband separation from the face would decrease as tightness increased was supported for the cavesson noseband both in the model horse head and in the live horse, although the difference between 0.5 and 0.0 finger-equivalents did not reach statistical significance at the maxillary and mandibular sites. When the Swedish noseband was tightened from 2 to 1.5 finger-equivalents, noseband separation did not change at any of the lateral locations but each further adjustment from 1.5 to 0.0 finger-equivalents was associated with decreased separation at all lateral locations. In general, the first hypothesis was supported.

These findings may reflect that the more rigid Swedish noseband needs greater longitudinal pre-tensioning than the more malleable cavesson. This finding highlights differences between individual nosebands and noseband types that should be considered in relation to an assessment of tightness. Further research is needed to determine the interaction between specific measuring tools and nosebands of different types.

The three lateral locations used in this study were selected on the basis of being easily identified without specialized anatomical knowledge and having minimal soft tissue between the skin and underlying bone. When comparing anatomical locations, the Swedish noseband showed no difference between the lateral nasal and maxillary locations at any noseband tightness. For the cavesson noseband at the 1.0 and 0.5 finger-equivalent adjustments, the maxilla had greater noseband separation than the lateral nasal site. However, the magnitude of the difference was <1.05 mm, which seems unlikely to be biologically significant. For the other noseband adjustments, there were no differences between the nasal and maxillary sites. Overall, these findings indicate a good level of equivalence between the two lateral locations on the skull.

The lateral maxilla is worthy of consideration as a potential lateral assessment location based on the results listed above, combined with the easy accessibility of the noseband at this site and the absence of aversive behaviours from the horses during the testing and positive feedback from the assessors, indicating that the maxilla was the preferred lateral location in 80% of horses. The nasal site was less popular with testers and 10% of horses showed aversive reactions at this site as tightness increased. The mandibular site was not popular with testers and the noseband separation data showed a different pattern than the two sites on the skull, especially for the Swedish noseband, which may have been related to the presence of the mandibular pad. These pads vary considerably among bridles, suggesting the mandibular site may be unsuitable due to a lack of consistency. When fitted with a cavesson noseband, the lateral mandibular values were higher than at the other two sites across all noseband adjustments. Interestingly, and reinforcing the complexities of bridle–horse interactions, the Swedish noseband separation values for the lateral mandible were significantly higher than for the lateral nasal site but did not differ from the maxillary site.

The horse’s head is an anatomically complex area of the body; its shape is not regularly geometric and it is subject to individual and breed variations [19]. In our study, we recruited a mixed population of breeds as representatives of the sport horse population [6]. Across the 96 horses measured with cavesson and Swedish nosebands adjusted from 2.0 to 0.5 finger-equivalent tightness, the CoV for the nasal and maxillary sites was “good” [12], but for the mandibular site the CoV was “acceptable”. These findings support the second hypothesis more strongly for the nasal and maxillary sites than for the mandibular site. The inclusion of a variety of breeds with different anatomical characteristics of their heads [19] is likely to have influenced the repeatability. At the mandibular site, the noseband’s proximity to the noseband fastening mechanism and the chin pad of the Swedish noseband are likely sources of increased variability. Based on these findings, the lateral mandible is not recommended as a suitable location for measuring noseband tightness.

One of the concerns with using a lateral location on the horse’s head to measure noseband tightness relates to the fact that the noseband fits most closely against the prominences of the nasal bones, the ventral mandibular rami and the premolar teeth. In the intervening areas, the noseband bridges across the bones. Consequently, the noseband fits more loosely and it is expected that noseband separation will be greater than that reported over the nasal bones at each tightness setting. A previous study reported an apparent relationship between frontal and lateral measurement of noseband forces with a slower rise in lateral forces as the noseband was tightened [10]. This study provides further data comparing noseband laxity at different anatomical locations, which is an important step forwards when informing the dimensions of a measuring tool. It should be noted that in some horses, the nasal bone in the midline nasal suture is in a groove/depression [19], which can cause inaccuracies; for example, by allowing the ISES tool to be admitted beneath the noseband at 0.0 finger tightness. Having additional locations where a calibrated measurement tool can be inserted beneath the noseband is seen as a positive step.

For our study, we used the ISES taper gauge, designed to simulate two, average-sized human fingers in a side-by-side orientation [4] to determine the noseband tightness level; this tool has been successfully used to measure noseband tightness in field-based and scientific studies [2,3,4,6,7,9]. For each noseband tightness adjustment, we measured the distance beneath the inner surface of the noseband and the skin at three anatomical locations on the lateral side of the head. Based on the data presented here, it is feasible to use a lateral site to measure noseband tightness if a measuring tool with appropriate dimensions were designed. The data from this study can be used to inform the dimensions of a lateral measuring tool.

A fundamental requirement for any measuring tool is that the design needs to be horse and steward/handler friendly, practical and safe to use. In our study, with the horses standing in stables or stalls, we found horses (46%, n = 40) displayed behaviours such as head raising/lowering, stepping forwards/backwards/sideways, left/right front limb pawing, and teeth grinding when the ISES taper gauge was used to determine the 2.0, 1.5, 1.0 and 0.5 finger-equivalents beneath the cavesson and Swedish nosebands. Our findings differ from those of other studies, where the majority of studied horses were reported to be “comfortable” when the ISES tool was used [2]. Differences among studies may arise due to the number of times that we admitted the ISES taper gauge beneath the noseband (i.e., once at a tack check vs. five times per noseband). Interestingly, a small percentage (10%) of horses displayed mildly aversive behaviour when the lateral nasal measurements were obtained, with this behaviour occurring at the tighter noseband adjustments (<1.0 finger-equivalents). The response of horses to noseband laxity is individual and while a minimum threshold for tightness is suggested [9,10], we recommend that riders should consider their horses’ behavioural responses in determining an appropriate noseband setting. Riders have a duty of care to train their horses using ethical approaches, and increasing noseband laxity in excess of the minimum threshold could improve equine welfare and riders should consider this in their decision-making [20,21].

## 5. Limitations

Inter-assessor repeatability was excellent in this study [12], but the force applied to the digital gauge to retract the noseband was not measured and may have varied between assessors. Whilst care was taken not to compress the soft tissues when the digital gauge was retracted, it is possible that a minimal amount of compression occurred. All nosebands and bridles were professionally fitted, so these data apply to correctly fitted bridles/nosebands. If the noseband is higher or lower than the “correct” position, it is likely to affect the distance between the noseband and the anatomical location, the ease of measurement and the values measured. Anatomical landmarks equivalent to those used for the cavesson and Swedish nosebands were not present in the more rostral position of the dropped noseband which precluded making lateral measurements. It has been reported that 65% of horses fitted with a dropped noseband displayed mildly aversive behaviour in response to insertion of the ISES taper gauge. These findings suggest that adaptations to the measuring tool may be needed to assess lateral tightness of nosebands that fit in different locations, e.g., dropped noseband, grackle noseband.

Only cavesson and Swedish nosebands are permitted with a double bridle and, although it is expected that they would be fitted consistently between bridle types, measurements should be repeated in horses wearing double bridles.

This study did not include a comprehensive behavioural assessment. It would be useful to study the horse’s behaviour and facial expressions while making measurements of different nosebands [22,23].

## 6. Conclusions

Three easily identified sites on the lateral aspect of the horse’s head were investigated for their suitability to assess noseband tightness. Sites on the lateral aspect of the nasal bone and the maxilla had comparable results at 2.0 and 1.5 finger-equivalent tightness. Their suitability for noseband tightness assessments were supported by finding the CoV to be “good” and the ROC AUC values (0.7–0.8) to be “acceptable” for determining a passing test for noseband tightness. No horses objected to the maxillary site, while 10% resisted at the nasal site, and the assessors reported the test was easily performed in these locations, especially the lateral maxilla. The mandible was a less suitable site for assessing noseband tightness due to showing a higher CoV for both nosebands at certain tightness levels; it was more affected by the presence of a chin pad on the Swedish noseband, and assessors reported it was more difficult make measurements on the mandible than more dorsally on the skull. Overall, it seems feasible to assess noseband tightness on the lateral side of the skull, with the lateral maxilla being the preferred site based on the data presented here. This information provides a basis for future studies.

## Figures and Tables

**Table 1:** Mean ± SD and coefficient of variation (CoV) from repeatability studies in which seven assessors measured the separation distance between the inner surface of the cavesson or Swedish noseband and the skin at three anatomical locations on a model horse and for three assessors performing the same measurements on three live horses. Dash (-) = measurements not obtained. SD: standard deviation; mm: millimetres; FET: finger-equivalents tightness.

		Cavesson Noseband			Swedish Noseband		
FET	Distance (mm) CoV (per Cent)	Lateral Nasal	Lateral Maxilla	Lateral Mandible	Lateral Nasal	Lateral Maxilla	Lateral Mandible
MODEL HORSE							
2.0	Distance CoV	25.2 ± 0.5 2%	26.9 ± 0.8 3%	26.5 ± 2.5 10%	-	-	-
1.5	Distance CoV	24.0 ± 0.7 3%	24.7 ± 1.4 6%	24.6 ± 1.3 5%	-	-	-
1.0	Distance CoV	20.9 ± 1.2 6%	21.4 ± 1.1 5%	21.1 ± 1.1 5%	-	-	-
0.5	Distance CoV	18.1 ± 0.7 4%	19.5 ± 1.8 9%	18.0 ± 0.6 4%	-	-	-
0.0	Distance CoV	16.9 ± 0.3 2%	18.4 ± 0.6 3%	14.4 ± 0.5 4%	-	-	-
3 LIVE HORSES							
2.0	Distance CoV	36.2 ± 0.4 1%	38.3 ± 0.8 2%	36.7 ± 1.1 3%	30.7 ± 1.1 4%	32.7 ± 2.5 8%	37.3 ± 1.8 5%
1.5	Distance CoV	34.2 ± 0.4 1%	36.7 ± 1.7 4%	34.7 ± 1.0 3%	27.8 ± 0.6 3%	28.7 ± 0.8 3%	33.5 ± 0.71 2%
1.0	Distance CoV	28.5 ± 1.1 4%	30.8 ± 1.0 3%	30.4 ± 1.7 6%	28.5 ± 0.8 3%	28.3 ± 0.7 1%	33.1 ± 0.4 1%
0.5	Distance CoV	24.8 ± 0.8 4%	25.4 ± 0.7 3%	24.1 ± 0.9 4%	26.1 ± 0.8 1%	27.6 ± 2.1 8%	29.3 ± 0.3 1%
0.0	Distance CoV	22.7 ± 0.4 2%	23.7 ± 0.6 2%	23.95 ± 0.5 2%	23.7 ± 0.5 2%	23.4 ± 0.6 3%	26.9 ± 0.7 2%

**Table 2:** Displaying the median threshold for 1.5 and 2.0 finger-equivalent tightness demonstrated acceptable ROC AUC values (0.7–0.8) for discrimination across the cohort for lateral nasal measurements of noseband tightness. F: finger-equivalent.

Median Threshold	ROC AUC 1.5 F Measurements		ROC AUC 2.0 F Measurements	
	Lateral Nasal Bone	Lateral Maxilla	Lateral Nasal Bone	Lateral Maxilla
1.5 F	ROC 1.0 (100%) CI: 1.0–1.0 PPV: 1.0 NPV: 0.1	ROC 1.0 (100%) CI: 1.0–1.0 PPV: 1.0 NPV: 0.1	ROC 0.78 (78%) CI: 0.74–0.82 PPV: 1.0 NPV: 0.1	ROC 1.0 (100%) CI: 1.0–1.0 PPV: 1.0 NPV: 0.1
2.0 F	ROC 0.76 (76%) CI: 0.72–0.80 PPV: 1.0 NPV: 0.1	ROC 1.00 (100%) CI: 1.0–1.0 PPV: 1.0 NPV: 0.1	ROC 1.00 (100%) CI: 1.0–1.0 PPV: 1.0 NPV: 0.1	ROC 1.0 (100%) CI: 1.0–1.0 PPV: 1.0 NPV: 0.1

## Data Availability

The original contributions presented in this study are included in the article. Further inquiries can be directed to the corresponding author.
